# Investigations into Flux-Free Plasma Brazing of Aluminum in a Local XHV-Atmosphere

**DOI:** 10.3390/ma15238292

**Published:** 2022-11-22

**Authors:** Jan Klett, Benedict Bongartz, Vincent Fabian Viebranz, David Kramer, Chentong Hao, Hans Jürgen Maier, Thomas Hassel

**Affiliations:** Institut für Werkstoffkunde (Materials Science), Leibniz Universität Hannover, 30823 Garbsen, Germany

**Keywords:** plasma brazing, plasma spectroscopy, local XHV-atmosphere, oxide layer, aluminum

## Abstract

As a lightweight construction material, aluminum plays a key role in weight reduction and, thus, sustainability in the transport industry. The brazing of aluminum and its alloys is impeded by the natural passivating oxide layer, which interferes with the brazing process. The presented study investigates the possibility of using a thermal silane-doped argon plasma to reduce this oxide layer in situ and thus eliminating the need to use hazardous chemical fluxes to enable high-quality brazing. Using plasma spectroscopy and an oxygen partial pressure probe, it was shown that a silane-doped argon plasma could significantly reduce the oxygen concentration around the plasma in a thermal plasma brazing process. Oxygen concentrations below 10^−16^ vol.-% were achieved. Additionally, metallographic analyses showed that the thickness of an artificially produced Al_2_O_3_-Layer on top of AlMg1 samples could be substantially reduced by more than 50%. With the oxide layer removed and inhibition of re-oxidation, silane-doped plasma brazing has the potential to become an economically efficient new joining method.

## 1. Introduction

Aluminum and its alloys represent an important class of construction material, increasingly used, e.g., in the automobile industry, where lightweight construction is seen as an important factor in increasing energy efficiency [1]. The high specific strength and thermomechanical characteristics make it valuable for automotive and especially aerospace applications [1,2]. Besides the low density, aluminum also shows good corrosion resistance due to its high oxygen affinity [3,4]. A dense passivating oxide layer (Al_2_O_3_) with a thickness of 2 nm to 5 nm is formed naturally on the metal’s surface, which prevents further oxidation [4,5]. This oxide layer is formed quickly and renews immediately after removal [5,6]. Thus, joining aluminum by welding or brazing creates challenges as the passivating oxide interferes with the joining processes [7,8].

The challenges can be summarized as follows:

- The poor electric conductivity of the oxide layer hinders the current transfer and stable arc formation for transferred arc processes like tungsten inert gas welding and other plasma processes.
- If the oxide layer is thermally destroyed (e.g., by an arc), the high reactivity of the metal with the atmosphere immediately leads to the formation of new oxide layers.
- Even small (250 to 500 ppmv) contaminations of oxygen in the inert shielding gas lead to the formation of new oxide layers.
- The oxide layer is thermodynamically very stable and has a higher melting point than the parent metal (*T*_m_(Al_2_O_3_) = 2072 °C vs. *T*_m_(Al) = 660 °C for pure aluminum). This leads to non-melted particles of the oxide layer suspended in the weld pool, which can prevent fusion (i.e., the binding of weld-joining partners) completely.
- The natural Al_2_O_3_ layer is hardly wetted by molten metals, which prevents proper bonding in brazing or soldering processes.

Even though the Al_2_O_3_ layer on aluminum workpieces is known to prevent diffusion processes between the base metal and filler material, and the low surface energy of the oxide layer even prevents the filler material from wetting the base metals surface [7,8], brazing is still considered a reliable and economical method for the joining of aluminum components [2,9,10]. While mechanical removal of the oxide layer is possible, it is not sufficient to allow effective brazing or welding since the surface will oxidize again immediately. Thus, chemical deoxidation using fluorides or chlorides is usually employed [11]. However, these chemicals are considered to be hazardous from an ecological as well as a health/safety perspective [12], especially in arc processes, where HCl and HF vapors can occur after chemical cleaning [13]. Researching alternative methods to remove the oxide layer is thus critical. Thus, the focus of this study was on the interrelation between the joint-surrounding oxygen, the oxide layer, and the weld pool. Specifically, a thermal plasma with a non-transferred arc was used. This method has the advantage that the electrode corrodes less, and interference of the oxide layer with the arc can be excluded. Plasma processes are widely used for modifying the surface properties (decontamination, hydrophilic properties, adhesion) of aluminum [4,14,15]. Plasma treatment can increase the surface energy and wettability of aluminum surfaces [16]. However, under atmospheric non-vacuum conditions, plasma treatment can also promote oxidation, leading to the formation of a thicker amorphous oxide layer (up to 15 nm with air used as a plasma gas) [4].

Since even small amounts of oxygen in the welding atmosphere (e.g., impurities in the shielding gas) can be crucial, the possibility of producing a local extreme-high vacuum (XHV, <10^−9^ Pa [17]) equivalent atmosphere (in terms of oxygen partial pressure) in the joining area was investigated. To achieve this goal, silane (SiH_4_) was locally added to the plasma gas. Silane is known to react quickly with oxygen and water vapor following Equations (1) and (2) [18]:

SiH_4_ + O_2_ → SiO_2_↓ + 2 H_2_ (1)

SiH_4_ + 2 H_2_O → SiO_2_↓ + 4 H_2_ (2)

In addition, a potential reaction between silane and aluminum oxide is:

(3) $$3\;{\mathrm{SiH}}_{4}+4\;{\mathrm{Al}}_{2}O_{3}\;\overset{\Delta T}{\to }\;3\;{\mathrm{SiO}}_{2}↓+6\;H_{2}O+8\;\mathrm{Al}$$

In order to analyze if this reaction can occur from a thermodynamical point of view, the free Gibbs energy is considered:

∆_f_*G*^°^ = ∆_f_*H*^°^ − *T*∆*S*^°^ (4)

where ∆_f_*G*^°^ is the standard molar Gibbs energy of formation in kJ∙mol^−1^, Δ_f_*H*^°^ is the standard enthalpy of formation in kJ∙mol^−1^, *T* is the temperature in K, and Δ*S*^°^ is the standard entropy in J∙mol^−1^∙K^−1^. By definition, if ∆_f_*G*^°^ is zero or negative, the reaction may occur under specific thermodynamic circumstances. The standard enthalpy of formation and the standard entropy of each component are shown in Table 1.

Thus, referring to Equation (4) and Table 1, the minimum necessary temperature to reduce the thickness of the aluminum oxide layer is 3281 K. In a thermal plasma (as used here), the heavy particles reach temperatures of 3 × 10^3^ K to 3 × 10^4^ K. Thermodynamic equilibrium is assumed, and therefore a uniform thermodynamic temperature can be considered. Variations in the real temperature distribution can be related to the state and parameters of the plasma, such as plasma gas type, plasma current and voltage [20,21,22]. Still, the thermodynamical consideration indicates that adding silane into a thermal plasma could theoretically reduce the thickness of an aluminum oxide layer. Prior studies already showed that very low oxygen contents in a process atmosphere could be achieved using silane [23,24]. If the oxide layer is removed by an ns-pulsed laser, a silane-doped atmosphere can prevent re-oxidation [24].

Hence, the hypothesis of the current study was that silane would reduce the oxygen- and vapor- fractions contained in the arc atmosphere to close to zero. Moreover, the existing oxide layer on the aluminum workpiece will be dispersed and vaporized by the hot plasma and will eventually react with the silane molecules. Finally, a local XHV-adequate oxygen-free atmosphere can be achieved, which actively prevents re-oxidation, and thus (combined with a locally oxide-free weld area) allows quality brazing or welding. If the oxide layer can be removed and re-oxidation is inhibited, silane-doped plasma brazing or welding has the potential to become an economically efficient new joining method.

## 2. Materials and Methods

To confirm or refute the hypothesis, experiments simulating a plasma-brazing process were performed in an argon atmosphere. A non-transferred argon plasma was ignited via a high-voltage impulse, starting the ionization. The plasma electrodes were placed 4 mm above a remotely handled, moveable aluminum workpiece mounted on a copper block for high thermal conduction. Silane was injected into the plasma atmosphere via a bypass nozzle. All experiments were performed firstly with a thermal argon plasma (reference) and secondly with a silane-doped thermal argon plasma. The natural oxide layer on aluminum is very thin (<2 nm). Unfortunately, in-situ XRD measurement is not feasible because the oxide layer shows an amorphous structure [4]. Other known methods to examine the thickness of the oxide layer, like X-ray photoelectron spectroscopy (XPS) or secondary ions mass spectrometry (SIMS), need the samples to stop reacting after the surface treatment in order to see if the natural oxide layer’s thickness could be reduced. Since the surface re-oxides very fast after the oxide layer is removed, said removal is hardly detectable afterward. Hence, samples with an artificially produced (anodized) oxide layer were examined instead.

Four analyses were performed in the present study:

- The oxygen partial pressure near the arc was measured in situ to determine if the silane reduces the oxygen content in the arc atmosphere.
- The light emitted by the plasma column was recorded by an emission spectrometer (1000 spectra in 4 s) to study changes, for example, in the hydrogen signal spectrum created by the reaction of silane and oxygen (Equations (1) and (2)).
- A workpiece (AlMg1 with 2 mm thickness) with an anodized aluminum oxide layer of 20 μm thickness was examined metallographically to determine if the thickness of the oxide layer could be reduced.
- The same workpieces (AlMg1; 2 mm thickness with 20 μm oxide layer) were analyzed via X-ray diffraction (XRD) to characterize surface adhesions.

**Figure 1:**
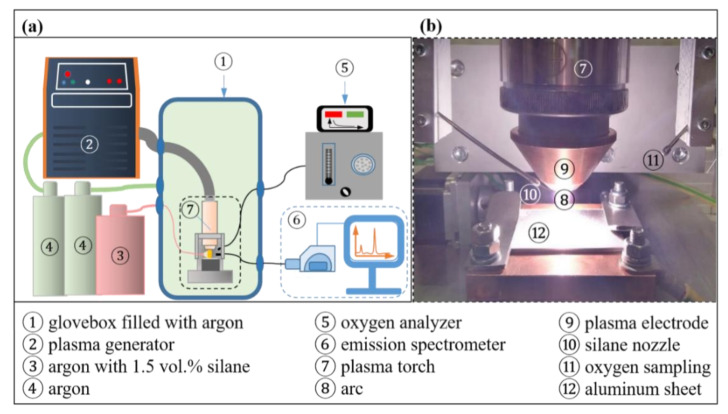
Experimental setup: (**a**) schematic representation of the glovebox with the surrounding analyzers and plasma equipment; (**b**) close-up view of the plasma zone.

All experiments were carried out in an argon-filled glove box (M. Braun Inertgas-Systeme GmbH, Garching bei München, Germany). The purity of the shielding gas was 99.998% Ar (ARCAL™ Prime from Air Liquide S.A., Paris, France). The glove box provided an enclosed test environment (Figure 1a). A HiFocus 160i plasma generator by Kjellberg Plasma und Maschinen GmbH (Finsterwalde, Germany) was used, generating a non-transferred DC plasma.

The gas creating the thermal plasma was 99.998% argon (ARCAL™ Prime from Air Liquide S.A., France, 99.998% Ar; flow rate 6.7 L/min). A bypass nozzle aimed at the arc (Figure 1b) allowed the addition of silane into the arc atmosphere. For this, argon mixed with 1.5 vol.-% silanes was used (silane UHP from Air Liquide S.A, France; flow rate 2.7 L/min). The oxygen partial pressure near the arc (the sensor was located 20 mm outside of the plasma column) was measured continuously using a VE02 oxygen analyzer (MESA GmbH, Filderstadt, Germany). For spectroscopic analyses, an emission spectrometer was used (Kymera 328i from Andor Technology Ltd., Belfast, UK). The software Andor Solis (Oxford Instruments plc, Abingdon, UK) was used to determine wavelength, intensity and full width at half maximum (FWHM) of every spectral line detected. To assess peak shifts in the spectrum caused by the instrument as well as possible errors of relative intensities, a calibration was performed before the measurements. The wavelength calibration of the spectrometer was done using the Hg-lines of a Pen-Ray line source (312.57 nm, 365.02 nm, 404.66 nm, 407.78 nm, 435.84 nm, 546.07 nm, 576.96 nm and 579.07 nm; Andor Technology Ltd., UK). The light was collected by a light collector with a 1 mm aperture. The gas supply, electrical cables and an optical fiber for spectroscopy were connected to the glove box via a bulkhead. XRD analyses were performed using a Discover D8 (Bruker AXS, Billerica, MA, USA) with a two-dimensional in an angular range from 40° 2Θ to 100° 2Θ using a spot diameter of 1 mm and 15° 2Θ incremental steps as well as Co-*K*_α_ radiation at a tube voltage of 45 kV and a tube current of 35 mA. The software DIFFRAC.EVA (Bruker AXS, USA) was used to generate the diffraction patterns from the measured data and to identify the phases present. Metallographic analyses were performed on cross-sections in accordance with ISO 17639:2022 (Destructive tests on welds in metallic materials—Macroscopic and microscopic examination of welds), without etchant.

**Figure 2:**
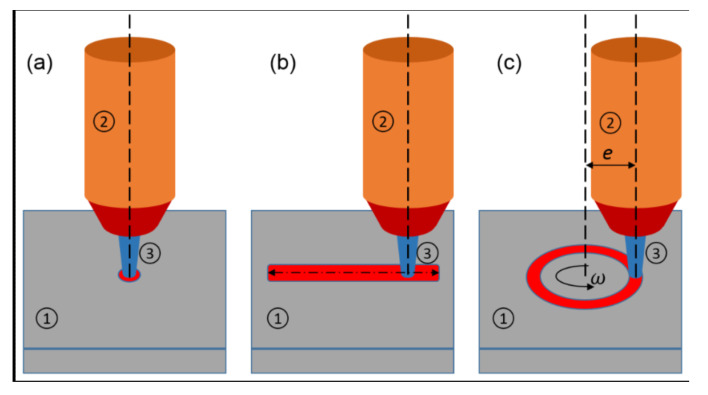
Schematic representation of the movement of the workpiece under the plasma torch: (**a**) punctual heating, (**b**) linear movement and (**c**) circular movement; (1) workpiece; (2) plasma torch; (3) arc; (*e*) horizontal distance of the plasma torch from the center of the circle; (ω) rotational velocity.

Preliminary experiments were carried out without moving the copper mount of the workpiece (punctual heating). This led to the melting of the aluminum beneath the oxide layer. Due to the low melting point of aluminum, the samples had to be moved relative to the plasma to prevent the substrate from melting. Since even with a moving sample, the sample surface melted after some treatment time, two different time/movement combinations were used: The first samples were moved linearly (bidirectional oscillating movement) in a straight line relative to the plasma torch at a speed of 1.9 mm/s. 24 s was identified as a safe time span to move the sample linearly under the thermal plasma without melting the samples’ surface. The second set of samples was rotated below the plasma. The rotation resulted in a circular track passed by the plasma (Figure 2), with every spot heated by the plasma being reheated with every new revolution. The horizontal distance of the plasma torch from the center of the circle was held constant at *e* = 10 mm. The rotational velocity (ω) was 1.67 rpm ≙ and 1.75 mm/s. This thermal treatment could be applied for 144 s without melting the sample. The vertical distance of the plasma torch (electrode) to the surface of the workpiece was kept constant at 4 mm for all experiments. A summary of the experiments performed in this study can be found in Table 2. The plasma parameters used for the experiments were:

- for punctual heating: 90 A, 29 V, 5 s;
- for linear movement: 70 A, 31 V, 24 s of plasma heating;
- for rotational movement: 80 A, 30 V, 144 s of plasma heating.

## 3. Results

### 3.1. Oxygen Concentration

**Figure 3:**
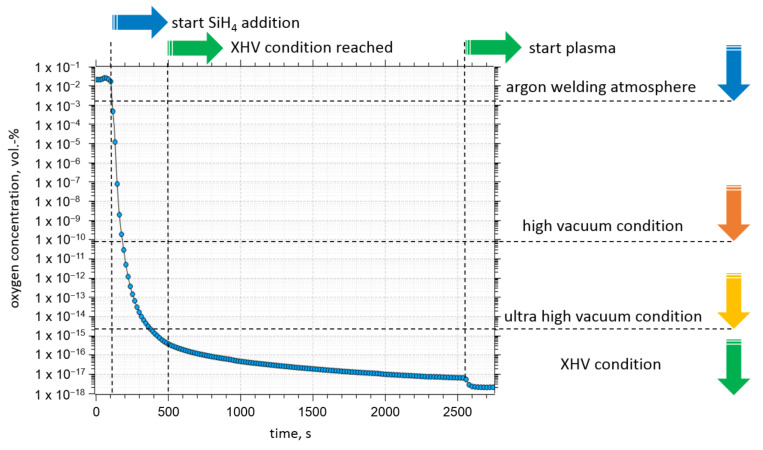
Evolution of the oxygen concentration measured close to the plasma during a typical Ar-silane plasma brazing experiment.

The results from a typical oxygen partial pressure measurement are shown in Figure 3.

The volume fraction of oxygen in the glovebox at the beginning of the experiments was 2 × 10^−2^ vol.-%. Adding silane to the plasma gas (60 s of silane injection) reduced the oxygen concentration to 1 × 10^−9^ vol.-% within 60 s. After 10 min, the volume fraction of oxygen in the gas mixture near the plasma could be locally reduced to 1 × 10^−16^ vol.-%, which, in terms of oxygen partial pressure, equals an XHV-atmosphere. The concentration begins to plateau after 30 min (approx. 1 × 10^−17^ vol.-%). Igniting the arc additionally reduces the oxygen partial pressure near the plasma, and the minimum oxygen concentration measured in all the experiments was 3 × 10^−20^ vol.-%.

### 3.2. Emission Spectroscopy of the Plasma

**Figure 4:**
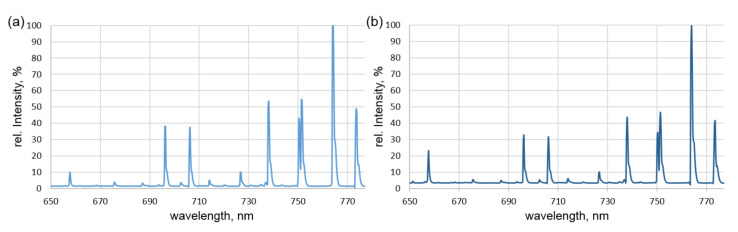
Recorded optical spectra with intensities normalized to the maximum peak at 663.51 nm (Ar-I) for (**a**) the thermal argon plasma; and (**b**) the thermal silane-argon plasma.

Representative results of the optical emission spectroscopy are displayed in Figure 4. The diagrams show the different peaks in the Ar and Ar-silane spectrum measured between the wavelengths 650 nm to 780 nm. The wavelengths of the significant peaks are 656.27 nm (H-I), 696.54 nm (Ar-I), 706.72 nm (Ar-I), 738.40 nm (Ar-I), 750.39 nm (Ar-I), 763.51 nm (Ar-I), and 772.40 nm (Ar-I). The peak with maximum intensity (in both cases, 763.51 nm (Ar-I)) was set as 100, and the other intensities were normalized accordingly to provide relative intensities.

**Figure 5:**
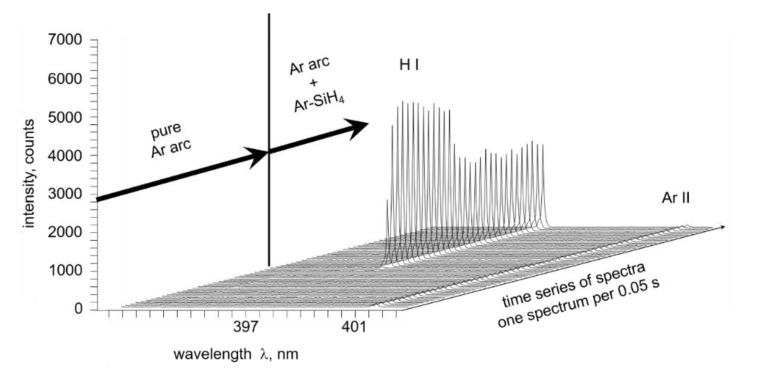
Continuously recorded optical spectra of the thermal plasma: silane injection at 9.4 s.

The changes in the spectra between 650 nm and 780 nm were used for further evaluation: Specifically, the relative intensities of single wavelength peaks in relation to other peaks within the same measurement were compared to the identical relation in other measurements to allow for a qualitative assessment regarding the evolution of the individual component contents. For instance, the peak of the characteristic hydrogen wavelength of 656.28 nm (H-I) was compared to the argon peak at 696.54 nm (Ar-I). Without the addition of silane, hydrogen should only be present due to residual moisture or as an impurity in the plasma gas. The ratio of the peaks (H/Ar) equaled 0.18 in the experiments without silane and 0.65 with the addition of silane to the plasma gas. Clearly, the addition of silane increased the amount of hydrogen in the plasma substantially. To further verify this finding, a continuous spectral measurement was performed (Figure 5). The focus was set on a different characteristic hydrogen spectral line (H-I at 397.01 nm) before and after adding silane to the plasma (continuous measurement). The intensity for this wavelength grew by more than 100 times when silane was added.

**Figure 6:**
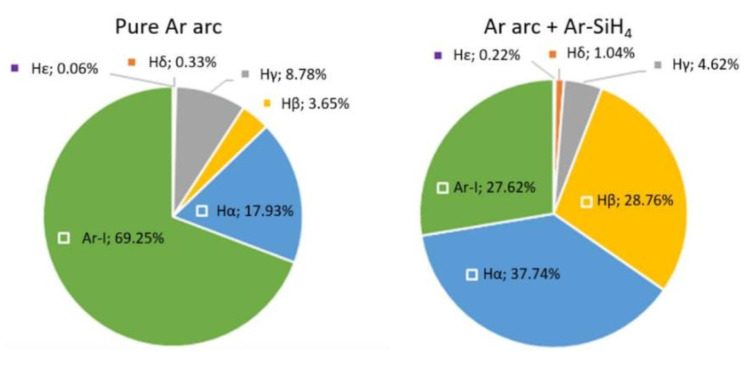
Plasma composition calculated from the spectral measurements; H-spectra lines follow the convention of Balmer [25]: Hα = 656.28 nm; Hβ = 486.13 nm; Hγ = 434.05 nm; Hδ = 410.17 nm; Hε = 397.01 nm.

From the whole spectrum, a relative composition of the plasma was calculated. The results are summarized in Figure 6.

### 3.3. Metallography of the Plasma-Treated Samples

**Figure 7:**
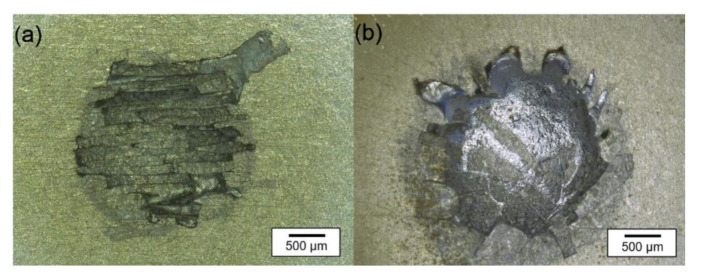
Photographs of the sample surface after 5 s of heating with the thermal plasma: (**a**) argon plasma; (**b**) argon + 1.5 vol.-% silane plasma.

Figure 7 shows the results of the preliminary trials with localized heating for 5 s at the same point (2 mm thick AlMg1 samples with an artificially produced anodic aluminum oxide layer). In this case, the spot actually hit by the plasma had a diameter of ≤1 mm, while the area of melted material after 5 s of heating reached up to 3 mm in diameter. For the argon plasma, Figure 7a shows a fractured oxide layer, while Figure 7b reveals the formation of a smoother surface without internal edges in the presence of the silane-doped plasma. Clearly defined edges can only be seen at the spots’ boundaries, where the plasma did not directly hit the surface and heating mainly happened by convection. It is concluded that in both cases, the aluminum was melted below the oxide surface. In the case without silane, the deformation of the aluminum substrate (due to the melting) led to the cracking of the oxide layer. The separated oxide chips then stayed on and covered the surface. Adding silane reduced the oxide layer before or while the aluminum melted.

**Figure 8:**
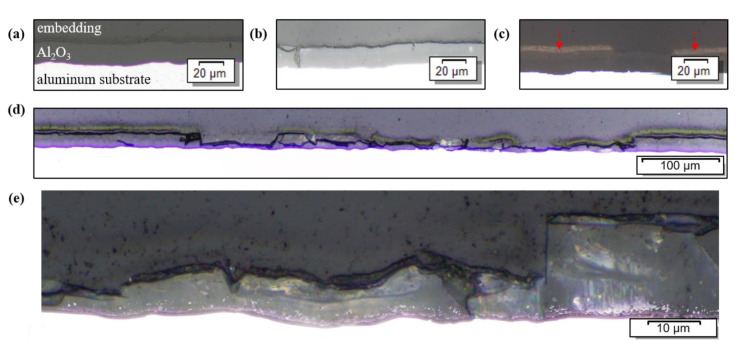
Micrographs of the cross-section of aluminum samples: (**a**) prior to the treatment with thermal plasma; (**b**) treated with thermal argon plasma (linear motion, 80 A, 24 s); (**c**) treated with thermal silane-argon plasma (linear motion, 80 A, 24 s); (**d**) treated with thermal silane-argon plasma (circular motion, 80 A, 144 s); (**e**) close up of (**d**) focusing on the difference between the initial oxygen layer (**right**) and the surface directly hit by the thermal silane-argon plasma (**left**).

Cross sections of the samples treated with the sample moving relatively to the plasma were polished and analyzed using optical microscopy (Stereomicroscope MZ 8 from Leica Microsystems GmbH, Wetzlar, Germany). The results are summarized in Figure 8.

With the linear movement of the workpiece under the pure argon thermal plasma, the thickness of the oxide layer could not be changed (Figure 8b). The addition of silane to the plasma upon linear motion did not significantly change the thickness of the oxide layer, either, in this test series (Figure 8c; reduction found is non-uniform and below 1 μm mean reduction). However, the circular movement of the sample, which allowed for a longer heating period (144 s vs. 24 s), resulted in a pronounced reduction of the aluminum oxide thickness (Figure 8d,e). The reduction occurred in the center of the heat-treated area (approximately 550 μm in width), where the plasma directly hit the surface. Since this reduction was not homogeneous (the artificial oxide layer was fractured), a mean value for the oxide layers thickness was measured at 20 spots, randomly distributed over the analyzed 550 μm wide surface area hit directly by the plasma. The mean thickness was reduced from 20 μm to 8.6 μm after 144 s of thermal treatment with a silane-doped argon plasma as compared to pure argon plasma. With 4 μm being the minimal oxide layer thickness achieved.

Besides the change in thickness of the aluminum oxide layer, the microscopic cross-sections of samples processed with silane also revealed a discontinuous grey-to-brown layer on the surface of the sample (Figure 8c, red arrows). These adhesions found on top of the aluminum oxide layer were further investigated (Section 3.4).

### 3.4. Analysis of the Surface Deposit

**Figure 9:**
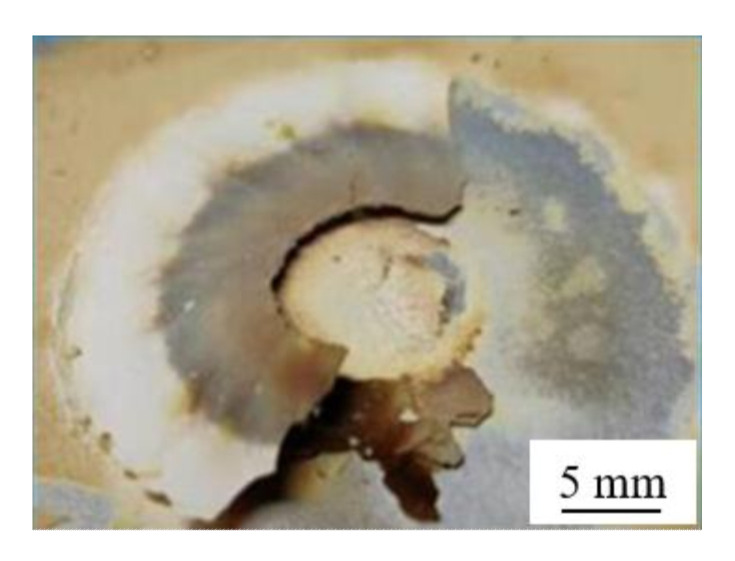
The surface deposit was found on samples after treatment with the thermal silane-argon plasma.

After the treatment with the thermal silane-argon plasma, a layer of yellow-brown deposit covered the surface of the aluminum samples (Figure 9). The deposit was friable, powdery and easy to remove.

The expected reaction between silane and aluminum oxide is shown in Equation (3). A thermal decomposition reaction of silane could occur in the thermal plasma as well [26]:

(5) $$5\;{\mathrm{Ar}}^{+}+{\mathrm{SiH}}_{4}+4\;e^{-}\overset{\Delta T}{\to }\;{\mathrm{Si}}^{+}+5\;\mathrm{Ar}+4\;H$$

**Figure 10:**
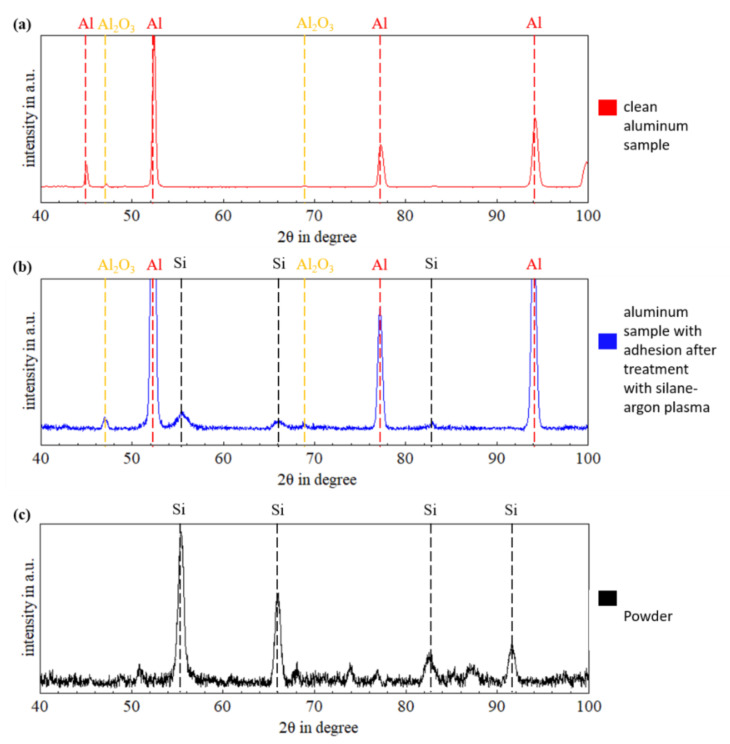
Diffraction patterns of (**a**) virgin aluminum sheet, (**b**) aluminum sheet with adhesions in the processing zone of the thermal silane-argon plasma and (**c**) powder collected from the processing zone affected by the thermal silane-argon plasma.

Thus, elemental silicon could form under reducing conditions and adhere to the aluminum/-oxide and affect the thermal plasma process. To verify this assumption, the surface deposit shown in the cross sections (Figure 8c, red arrows) was further examined using X-ray diffraction (XRD). The results are shown in Figure 10. The virgin aluminum sample solely shows diffraction peaks of aluminum and aluminum oxide, as expected (Figure 10a). The oxide peaks feature a low intensity. After the thermal treatment with the silane-argon plasma, the sample’s surface showed diffraction peaks of elemental silicon (Figure 10b). This fits the assumption that the thermal decomposition reaction of silane in the thermal plasma resulted in crystalline silicon (Equation (5)). This can also be seen in the measurement results of the pure powder (surface deposit brushed from the surface of the sample) (Figure 10c), where only the silicon peaks are present.

## 4. Discussion

The results of the oxygen measurement show that XHV-adequate oxygen concentrations can locally be achieved by adding silane to the arc process zone. This is similar to earlier results in related work [23,24]. The spectroscopic analyses are suitable to further elaborate the mechanisms leading to the previously found oxygen reduction. Both the change of the argon-hydrogen relation (comparison of peaks in different measurements), as well as the rise of the hydrogen peak after the addition of silane in the continuous measurement, illustrate the formation of hydrogen when silane is injected into the arc zone i.e., hydrogen originates from the silane (SiH_4_) and is freed by the reaction of silane with oxygen (or water), in which silane reacts to SiO_2_, cf. Equations (1) and (2) [18].

These findings inevitably lead to a potential risk that needs to be assessed: In thermal arc-joining processes, gaseous hydrogen always has to be monitored carefully. Since the heat of an arc is sufficient to dissociate molecular hydrogen into hydrogen atoms or ionized hydrogen [27,28], the hydrogen released by the silane reaction could be dissolved by melted base- or filler-metal [29,30]. Aluminum is susceptible to hydrogen embrittlement and is thus at risk if the joining process introduces excessive amounts of hydrogen to the joining area [31,32]. The influence of the hydrogen and the critical hydrogen concentrations to ensure joints without hydrogen embrittlement has to be investigated further. Given the different processing conditions, this has to be done separately for the brazing and welding processes. Besides the risk of embrittlement, the addition of hydrogen can also raise the temperature in an argon plasma and can, in consequence, alter the heat input in a welding or brazing process [33,34,35], which should also be investigated.

The existence of silicon (Si) in the form of surface adhesions, as detected by the XRD analysis, indicates that there was excess silane added to the plasma, i.e., more than needed to just reduce the atmospheric oxygen. Based on the stoichiometric reactions (Equations (1) and (2)), only amorphous silicon dioxide (SiO_2_) should appear. The existence of pure silicon demonstrates that there was more silane present than oxygen to react with. While this shows how effective the local oxygen reduction worked, it could also cause challenges if the silicone interferes with the joining process or parts of the silicon powder are dissolved/suspended in melted base- or filler metal. Given the needs of the solar panel industry, the synthesis of (elemental) silicon nano powder or amorphous hydrogenated silicon from silane is well-known and researched [36,37]. Investigations of the plasma-enhanced chemical vapor deposition (PE-CVD) process showed that using plasma, the conversion rate of the decomposition of silane into silicon, as well as the deposition and the crystallinity of the silicon, depend on the input power, the diluent gas, the total gas pressure, the quenching rate, the substrate temperature, and the flow rate of the silane [36,37]. The applicability of those results on silane-doped plasma brazing/welding should be investigated to optimize the process conditions.

Both factors, excessive hydrogen content and residual silicon adhesions, can be reduced if the amount of silane added to the joining process is lowered. There has to be enough silane in the process atmosphere to react with the oxygen (Equations (1) and (2)). However, not only the atmospheric oxygen has to be considered when the amount of silane needed is calculated. As the metallographic analysis showed, the combination of thermal plasma and silane enables the reduction of a surface oxide layer. The released oxygen has to be reduced by the silane, too. In the present study, this reduction worked very well when the sample was moved below the plasma for an extended time period (144 s). This duration, however, was only possible (without melting the aluminum substrate) by rotating the sample and thus creating a long circular path for the thermal plasma to reduce the thick anodized oxide layer. In this case, the thickness of this artificially produced Al_2_O_3_layer was reduced by more than 50% (>10 μm mean reduction). This reduction is an important achievement since prior studies showed that a silane-free plasma treatment could also promote oxidation, leading to the formation of a thicker amorphous oxide layer [4]. Considering that the natural oxide layer on aluminum is only 2 nm thick, the reduction effect achieved in the present study is more than sufficient. Previous studies already showed that when the oxide layer is removed and the joining atmosphere prevents re-oxidation, wetting of the surface is no challenge anymore, and flux-free brazing is possible [7,12,24]. This effect can be exploited for the plasma process, too. Moreover, studies suggest that plasma processes could even increase the surface energy and thus the wettability [14,16].

Summarizing the results, the initial hypothesis stated in the introduction can be confirmed: The addition of silane to the plasma gas reduced the oxygen contained in the arc atmosphere to XHV-adequate oxygen levels. The oxide layer on an aluminum workpiece can be dispersed and vaporized by a thermal plasma, and the oxygen released will react with the silane. The local XHV-adequate oxygen-free atmosphere prevents re-oxidation, and thus (combined with a locally oxide-free brazing area) makes a high-quality flux-free plasma brazing process possible. Work is underway to study the effects this new approach has on the mechanical properties of the joint.

## 5. Conclusions

The present study investigated the possibility of creating a local XHV-adequate oxygen-free plasma atmosphere through the addition of silane.

The main findings can be summarized as follows:

- Adding silane to the thermal arc plasma resulted in a significant reduction (Δ > 10^14^) of the oxygen partial pressure near and in the plasma.
- The spectroscopic analyses confirmed the reaction of silane and oxygen under the formation of hydrogen, silicon oxide and elemental silicon.
- The thickness of an artificially produced oxide layer could be reduced (>50%) by adding silane to the thermal plasma.
- Although the silane-doped thermal plasma reduced the oxide layer, the release of hydrogen and the formation of an elemental silicon layer on the aluminum workpiece are issues that might affect the quality of brazed or welded joints.

## Figures and Tables

**Table 1:** Standard thermodynamic data for the materials considered [19].

Material	Enthalpy, kJ∙mol^−1^	Entropy, J∙mol^−1^∙K^−1^
Al_2_O_3_ (s)	−1656.864	59.8312
SiH_4_ (g)	30.5432	204.51392
H_2_O (g)	−241.818464	188.715136
SiO_2_ (s)	−903.49296	46.8608
Al (s)	0	28.32568

**Table 2:** Summary of the experiments performed in this study.

Experiment	Aim	Sample	Analysis
quantitative oxygen concentration near the plasma	varify atmosphere-oxygen reduction	NA	oxygen partial pressure sensor
qualitative hydrogen concentration in the plasma	varify silane reaction	NA	emission spectrometer
punctual plasma heating	reducing the thickness of the oxide-layer	AlMg1; 2 mm thickness with 20 μm anodized oxide layer	metallography
linear heating	reducing the thickness of the oxide-layer	AlMg1; 2 mm thickness with 20 μm anodized oxide layer	metallography
circular heating	reducing the thickness of the oxide-layer	AlMg1; 2 mm thickness with 20 μm anodized oxide layer	metallography
additional surface examination	identification of surface adhesions	AlMg1; 2 mm thickness with 20 μm anodized oxide layer	XRD

## Data Availability

Data are available upon request.
